# Editorial: Neurocognitive dysfunction in people living with HIV and the underlying brain mechanisms

**DOI:** 10.3389/fneur.2025.1674176

**Published:** 2025-08-15

**Authors:** Wei Wang, Zhongkai Zhou, Yuxin Shi, Puxuan Lu, Hongjun Li

**Affiliations:** ^1^Henan Clinical Research Center of Infectious Diseases (AIDS), Affiliated Infectious Diseases Hospital of Zhengzhou University, Henan Infectious Diseases Hospital, Zhengzhou, China; ^2^Department of Radiology, Beijing Youan Hospital, Capital Medical University, Beijing, China; ^3^Shanghai Public Health Clinical Center, Fudan University, Shanghai, China; ^4^Shenzhen Center for Chronic Disease Control, Shenzhen, China

**Keywords:** HIV-associated neurocognitive disorders, asymptomatic neurocognitive impairment, multimodal neuroimaging, precision neuroscience, exosome neuroimaging integration

With the widespread implementation of combination antiretroviral therapy (cART), HIV infection has evolved into a manageable chronic condition, with life expectancy significantly extended—approaching that of the general population in some cases (1). However, the central nervous system, as an “immune-privileged site” and potential viral reservoir, may harbor residual virus and persistent inflammation even under effective plasma viral suppression (2, 3) Studies show that neuronal injury can still occur in people living with HIV in the cART era, driven by factors including viral protein neurotoxicity, microglial activation, glutamate excitotoxicity, immune dysregulation, comorbidities, and antiretroviral neurotoxicity (4, 5).

HIV-associated neurocognitive disorders (HAND) have thus emerged as a major challenge in chronic disease management, with a prevalence ranging from 30% to 50% (6, 7). The milder forms of HAND—namely, asymptomatic neurocognitive impairment (ANI) and mild neurocognitive disorder—are the most common. Despite subtle or absent clinical symptoms, they may reflect early neurodegenerative processes (7, 8). This Research Topic features eight representative studies and reviews published in the Frontiers series, spanning structural normalization, local cognitive norms, brain network changes, bibliometric trends, multimodal imaging, and exosome integration—together offering a comprehensive overview of recent progress and future directions in HAND research.

Recent studies have emphasized optimizing structural imaging normalization and cognitive reference systems to enhance early HAND detection. Nguchu et al. proposed that ventricular volume (VV), compared to the conventional intracranial volume, serves as a more suitable normalization metric for structural MRI, as it more sensitively captures basal ganglia and limbic system atrophy in people living with HIV—particularly in detecting interactions with aging. VV-based correction holds promise for enhancing structural evaluation in populations at risk for HAND. In a population-based study in China, Chen C. et al. demonstrated that applying locally derived cognitive norms—rather than international references—enabled more sensitive detection of attention and memory deficits in the ANI stage, particularly in immediate recall tasks, thereby reducing potential misclassifications related to cultural or educational background differences.

The brain is increasingly viewed as a complex, interconnected network, warranting analysis from a systems perspective. In a 1.5-year longitudinal study of individuals with HIV at the ANI stage, Xu et al. employed voxel-based morphometry and structural connectivity network approaches. They found baseline reductions in gray matter volume in the right middle temporal gyrus and left middle frontal gyrus, along with decreased hubness in the anterior cingulate cortex. During follow-up, additional impairments were observed in the volume of the right fusiform gyrus and the nodal efficiency of the inferior frontal gyrus. Although the global small-world topology remained preserved, the progressive degradation of key regional nodes suggests a cumulative vulnerability of the brain network—potentially reflecting early trajectories of HAND progression.

Against the backdrop of expanding multidimensional research, systematically mapping the developmental trajectory and thematic evolution of HAND studies is essential for clarifying future research priorities. Zhou T. et al. applied CiteSpace to perform a comprehensive visual bibliometric analysis of HAND-related publications from 2000 to 2023. Their findings revealed a three-phase thematic evolution: an early focus on direct neurotoxic mechanisms of HIV, a mid-phase emphasis on chronic inflammation and immune activation, and a recent shift toward gut–brain axis dysfunction, exosome-mediated signaling, and metabolic abnormalities. Keyword burst analysis showed that terms such as “biomarkers,” “metabolic disorders,” and “exosomes” have recently surged in frequency, indicating a growing emphasis on mechanistic targeting and precision intervention. The authors further noted that while international collaborations are currently centered around the United States, United Kingdom, and South Africa, Chinese research teams are increasingly emerging on the global stage. Multicenter studies incorporating artificial intelligence and multi-omics integration are becoming the dominant research paradigm.

Multimodal imaging has shown synergistic value for HAND identification and mechanistic analysis. Chen J. et al. reviewed EEG-fMRI integration, noting its ability to capture both temporal and spatial neural activity—particularly sensitive to disruptions between the default mode network and anterior cingulate cortex. Zhou Z. et al. proposed a “multimodal connectomics” framework that integrates structural MRI (sMRI), diffusion tensor imaging (DTI), and functional MRI (fMRI) data to uncover network damage patterns in HAND using graph-theoretical metrics and machine learning techniques, offering a novel path for subtype stratification and mechanistic decoding. Wang et al. provided a comprehensive review of recent advances in multimodal imaging techniques—including sMRI, fMRI, DTI, magnetic resonance spectroscopy, and arterial spin labeling—in the study of HAND. They emphasized that these modalities enable quantification of brain atrophy, white matter injury, and metabolic abnormalities in people living with HIV. The authors further emphasized that incorporating peripheral biomarkers, such as neurofilament light chain (NfL), could enhance the diagnostic specificity of HAND. They advocated for greater standardization of preprocessing pipelines, harmonization of atlas selection, expansion of longitudinal cohorts, and the integration of AI and imaging–genomics approaches to accelerate the transition toward precision neuroimaging in HAND research.

Beyond advancing neuroimaging research, mechanistic exploration of HAND is gradually extending toward a central–peripheral integrative perspective. Luo et al. introduced the concept of a “brain–blood bridge” and systematically reviewed the synergistic potential of neuroimaging and exosome analysis in elucidating HAND pathophysiology. Neuron-derived exosomes (e.g., tau, miR-146a) are capable of crossing a compromised blood–brain barrier, and fluctuations in their levels are closely associated with imaging markers such as functional connectivity and gray matter integrity, reflecting processes of neuroinflammation and synaptic injury. The authors categorized three potential central–peripheral coupling mechanisms and highlighted key challenges, including a lack of methodological standardization and limited dynamic monitoring capabilities. They propose that future studies could benefit from establishing a “neuroimaging–exosome feature map” to decode coupling mechanisms in HAND, thereby advancing early detection and precision intervention.

HAND research is entering a new phase characterized by the convergence of network visualization, multimodal integration, and fluid biomarker synergy. Future directions include leveraging ultra-high-field MRI, portable EEG systems, artificial intelligence algorithms, and peripheral omics platforms to facilitate early detection, subtype classification, and mechanism-based interventions for HAND. These advances promise to usher in a new era of precision neuroscience for the neurocognitive care of people living with HIV.
